# Synergistic Immediate Cortical Activation on Mirror Visual Feedback Combined With a Soft Robotic Bilateral Hand Rehabilitation System: A Functional Near Infrared Spectroscopy Study

**DOI:** 10.3389/fnins.2022.807045

**Published:** 2022-02-04

**Authors:** Yaxian Qiu, Yuxin Zheng, Yawen Liu, Wenxi Luo, Rongwei Du, Junjie Liang, Anniwaer Yilifate, Yaoyao You, Yongchun Jiang, Jiahui Zhang, Aijia Chen, Yanni Zhang, Siqi Huang, Benguo Wang, Haining Ou, Qiang Lin

**Affiliations:** ^1^Department of Rehabilitation, The Fifth Affiliated Hospital of Guangzhou Medical University, Guangzhou, China; ^2^Department of Rehabilitation, Guangzhou Medical University, Guangzhou, China; ^3^Department of Rehabilitation, Longgang District People’s Hospital of Shenzhen, Shenzhen, China; ^4^Department of Rehabilitation, The Third Affiliated Hospital of The Chinese University of Hong Kong, Shenzhen, China

**Keywords:** mirror visual feedback, soft robotic bilateral hand rehabilitation system, functional near infrared spectroscopy, cortical activation, synergistic gain effect

## Abstract

**Background:**

Mirror visual feedback (MVF) has been widely used in neurological rehabilitation. Due to the potential gain effect of the MVF combination therapy, the related mechanisms still need be further analyzed.

**Methods:**

Our self-controlled study recruited 20 healthy subjects (age 22.150 ± 2.661 years) were asked to perform four different visual feedback tasks with simultaneous functional near infrared spectroscopy (fNIRS) monitoring. The right hand of the subjects was set as the active hand (performing active movement), and the left hand was set as the observation hand (static or performing passive movement under soft robotic bilateral hand rehabilitation system). The four VF tasks were designed as RVF Task (real visual feedback task), MVF task (mirror visual feedback task), BRM task (bilateral robotic movement task), and MVF + BRM task (Mirror visual feedback combined with bilateral robotic movement task).

**Results:**

The beta value of the right pre-motor cortex (PMC) of MVF task was significantly higher than the RVF task (RVF task: -0.015 ± 0.029, MVF task: 0.011 ± 0.033, *P* = 0.033). The beta value right primary sensorimotor cortex (SM1) in MVF + BRM task was significantly higher than MVF task (MVF task: 0.006 ± 0.040, MVF + BRM task: 0.037 ± 0.036, *P* = 0.016).

**Conclusion:**

Our study used the synchronous fNIRS to compare the immediate hemodynamics cortical activation of four visual feedback tasks in healthy subjects. The results showed the synergistic gain effect on cortical activation from MVF combined with a soft robotic bilateral hand rehabilitation system for the first time, which could be used to guide the clinical application and the future studies.

## Introduction

Mirror therapy or mirror visual feedback (MVF) was first proposed by Ramachandran et al. (1995) and applied to the treatment of limb phantom pain. It was found that the input of a mirrored visual illusion could activate brain plasticity of the affected limb and thus inhibit the abnormal displacement of the central functional area of the brain and in turn alleviate pain (Ramachandran and Altschuler, 2009; Herrador Colmenero et al., 2018). Subsequently, MVF has been widely used in neurological rehabilitation. Thieme et al. (2018) meta-analysis found moderate quality of evidence that MVF was beneficial in improving motor dysfunction and activities of daily living. Until now, the relevant mechanistic hypotheses involved in MVF mainly include the following three aspects: MVF can activate the mirror neuron system, thus inducing or enhancing motor imagery (Grèzes and Decety, 2001); MVF might also conducive to the recruitment of ipsilateral cortical spinal cord bundles (Zheng and Hu, 2011) and can enhance attention in affected sides via a mirror illusion, thus activating the motor network in the brain region of the affected side (Deconinck et al., 2015). However, due to the small sample size and large heterogeneity of patients (including the locations and side of brain injury, motor and cognitive impairment levels, sensory function, and even age, etc.) (Luo et al., 2020), the clinical efficacy and related mechanisms of MVF still needs further analysis. More recently, the Central-Peripheral-Central closed-loop regulation mode, formed by the combination of MVF as a central intervention method with other peripheral interventions, has become a research hotspot to further improve the technique. In recent years, some MVF studies have combined it with electromyographic biofeedback (Kim and Lee, 2015; Liu et al., 2021), EMG-triggered electrical stimulation (Schick et al., 2017), electrical stimulation (Lee and Lee, 2019), robot-assisted therapy (Ji-feng et al., 2019), and other intervention methods. These results suggest that combination therapy has potential for improving moderate to severe upper limb dysfunction after stroke. In the past some studies simply superimposed MVF and other interventions in order [such as MVF combined with acupuncture (Yin et al., 2020), robot-assisted therapy (Thakkar et al., 2020; Rong et al., 2021)], and failed to form MVF synchronized with other peripheral interventions to achieve central-peripheral-central closed-loop measures. Additionally, although some studies (such as MVF synchronous electromyographic biofeedback, EMG-induced electrical stimulation) have achieved closed-loop intervention formally, the muscle electrical stimulation given by the electrode could not accurately induce hand movement resulting in a significant difference between the passive movement of the affected side and the mirror movement leading to the poor effect of the closed-loop regulation mode. Therefore, it is necessary to explore a better MVF synchronous intervention mode to further improve the effect of closed-loop regulation.

In our study we use a soft robotic bilateral hand rehabilitation system. The soft robotic bilateral hand rehabilitation system is a pneumatically driven soft robot based on bilateral hand movement intervention for patients with hand dysfunction after stroke. In this method, the unaffected hand is fitted with an inductive glove for normal hand movement, while the affected hand is fitted with an exoskeleton robotic glove for passive hand movement, imitating the unaffected hand. The principle is to have the inductive glove record normal hand movements of the unaffected hand and input data into the exoskeleton robotic glove to guide the affected hand to simulate movement. Therefore, it is also defined as robot – mediated bilateral therapy (Haghshenas-Jaryani et al., 2020). This intervention method may be related to three potential principles of nerve remodeling. First, soft robotic gloves can not only activate the primary motor cortex of the affected side by driving the affected hand through the unaffected hand, but also trigger the proprioceptive input by moving the affected hand, so as to activate the corresponding primary sensory cortex (Haghshenas-Jaryani et al., 2019), and establish effective “peripheral” stimulation feedback to “central.” Second, soft robotic gloves realize bilateral exercise training mode through the joint action of the unaffected hand, which is conducive to the normalization of inter-cortical inhibition between cerebral hemispheres. Previous studies have shown that the mechanism of bilateral movement pattern was that the simultaneous movement of the same muscle groups on both sides was beneficial to the activation of similar neural networks in bilateral hemispheres, thereby reducing inter-hemispheric inhibition and improving the functional performance of paralytic hand (Renner et al., 2020). Several studies showed that bilateral training was superior to neurodevelopment treatment and unilateral robot-assisted training in improving upper limb motor function after stroke (Chen et al., 2019; Straudi et al., 2020). Finally, the linkage device of the robotic glove make it easy to realize the repeated movement of the affected hand, which can continuously provide positive feedback to the central nervous system through the peripheral movement and strengthen the neuronal circuit (Mane et al., 2020), thus facilitating the neural remodeling of the brain on the affected side. Moderate-quality evidence showed a beneficial effect of high repetitive task practice is considered to be an effective intervention method (Pollock et al., 2014). Repeated exercise is considered to be the physiological basis for motor learning, and motor and sensory coupling contributes to the adaptation and recovery of neural pathways (French et al., 2007). Overall, soft robotic gloves can provide positive and effective “peripheral” stimulation, and MVF is based on the mechanism of motor imagery and mirror neurons to produce central regulation. If they are combined with synchronous intervention, a complete and strong “central-peripheral-central” closed-loop regulation can be theoretically formed. However, whether it can bring a better clinical effect and its mechanism remain unclear.

Previous studies on central nervous regulation based on MVF mostly used functional magnetic resonance imaging (fMRI) (Rjosk et al., 2017; Manuweera et al., 2018), electroencephalography (EEG) (Al-Wasity et al., 2019; Luo et al., 2020; Fong et al., 2021) and functional near infrared spectroscopy (fNIRS) (Inagaki et al., 2019; Bai et al., 2020), but these studies are limited by the high cost of fMRI examination, spatial limitations, and low spatial resolution of EEG. fNIRS is portable, environmentally friendly, and has relatively high spatial resolution. This study included healthy young subjects to minimize the differences between individuals, such as part of brain damage, degree of surviving motor function and cognitive function, and used synchronous fNIRS to determine whether MVF and a soft robotic bilateral hand rehabilitation system have synergistic effects on cortical activation. The results can guide future clinical treatments and mechanistic research.

## Materials and Methods

### Participants

Our self-controlled study recruited 20 healthy subjects from Guangzhou Medical University who met the following criteria: (a) 18–35 years old; (b) right hand dominant according to the Edinburgh Handedness Inventory; (c) no previous affected vision field or vision diseases; (d) no central nervous system diseases; and (e) no upper limb fracture history and upper limb deformity. The study was approved by the Ethics Review Committee of the Fifth Affiliated Hospital of Guangzhou Medical University (No. GYWY-L2021-74). All subjects signed informed consent forms. This study was also approved by the China Clinical Registration Center (No. ChiCTR2100052042)^1^.

### Procedure and Experimental Tasks

All recruited subjects were asked to perform four different visual feedback tasks with simultaneous fNIRS monitoring. The order of tasks was random and based on random computer software. The right hand of the subjects was set as the active hand (performing active movement), and the left hand was set as the observation hand (static or performing passive movement under soft robotic bilateral hand rehabilitation system). The active hand followed the preset sound prompt to grasp or extend at a frequency of 0.5 Hz.

Mirror visual feedback used a three-dimensional triangular mirror box with an area of 30 cm × 30 cm. The soft robotic bilateral hand rehabilitation system contains soft-robotic bilateral gloves (SY-HR06, Siyi Intelligent Technology Co., Ltd., Shanghai, China) including one for the motion-command glove on the right hand (active side) and the other for the motion-actuator glove on the left hand (slave side). Through this master-slave configuration, the motion trajectory of the active hand (right, master) provides input instructions for robotic glove that drive the passive limb (left, slave) to perform synchronous motion. All subjects were asked to wear soft-robotic bilateral gloves to maintain the same cortex activation from glove itself during four tasks. Before testing, the robotic gloves should be calibrated to confirm correct mirror motion.

**FIGURE 1:**
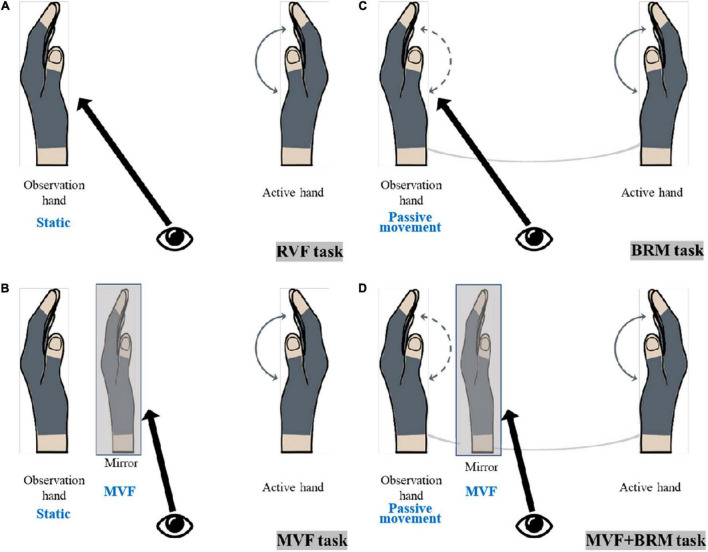
The diagram of visual feedback tasks. **(A)** RVF Task (real visual feedback task); **(B)** MVF task (mirror visual feedback task); **(C)** BRM task (bilateral robotic movement task); **(D)** MVF + BRM task (Mirror visual feedback combined with bilateral robotic movement task).

According to whether the observed hand was in static or passive movement through linkage, the VF tasks could be divided into a non-linkage state (real visual feedback task, RVF task; mirror visual feedback task, MVF task) and linkage state (bilateral robotic movement, BRM task; MVF + BRM task). They could also be divided based whether the mirror image was involved: real visual feedback from the observed hand (real visual feedback task, RVF task; bilateral robotic movement, BRM task) and mirror visual feedback from active hand (mirror visual feedback task, MVF task; MVF + BRM task). The four VF tasks were designed as follows (Figure 1):

(1) RVF task: The active hand performed grasping/extension without mirror visual feedback; the observed hand remained static, and visual feedback was from the observed hand (Figure 1A).
(2) MVF task: The active hand performed grasping/extension with mirror visual feedback; the observed hand remained static, and visual feedback was from the mirror illusion of the active hand (Figure 1B).
(3) BRM task: The active hand performed grasping/extension without mirror visual feedback; the observed hand performed passive movement in the soft robotic bilateral hand rehabilitation system; visual feedback was from the observed hand (Figure 1C).
(4) MVF + BRM task: The active hand performed grasping/extension with mirror visual feedback; the observed hand performed passive movement under soft robotic bilateral hand rehabilitation system, and visual feedback was from the mirror illusion of the active hand (Figure 1D).

### Functional Near Infrared Spectroscopy Measurement

#### Data Acquisition

**FIGURE 2:**
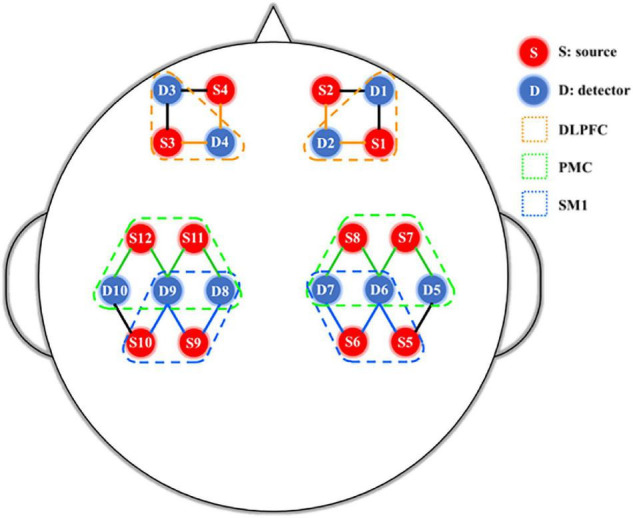
fNIRS 24-channel placement. SM1, primary sensorimotor cortex; PMC, pre-motor cortex; DLPFC, dorsolateral prefrontal cortex.

Synchronized fNIRS (NirSmart, Danyang Huichuang Medical Equipment Co., Ltd., Beijing, China) was used to monitor hemodynamic responses during different visual feedback tasks. 12 source probes and 10 detectors were integrated into a custom head cap that was fixed on the subjects’ heads and adjusted according to size and shape. Two infrared wavelengths (730 and 850 nm) emitted by the transmitter were received by a pair of adjacent detectors with an interval of 30 mm, and were collected at a sampling rate of 10 Hz. The source probe and detector could form 24 channels according to the placement of the 10–20 systems (Chatrian et al., 1988; Oostenveld and Praamstra, 2001; Fornia et al., 2020a). The position of the source probe and detector in this study were shown in Figure 2. After subject preparation ready, a fNIRS gain quality check was performed before the test to ensure that the data acquisition was neither under-gained nor over-gained.

**FIGURE 3:**
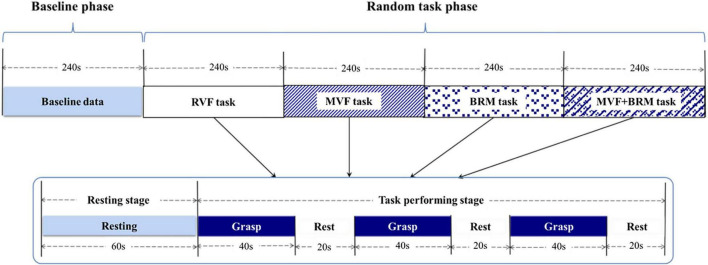
fNIRS testing procedure including baseline phase and visual feedback task phase. The order of four tasks were randomized. Each visual feedback task consists of three trials. RVF task, real visual feedback task; MVF task, mirror visual feedback task; BRM task, bilateral robotic movement task; MVF + BRM task, Mirror visual feedback combined with bilateral robotic movement task.

The fNIRS testing was divided into a baseline phase (240 s) and a random task phase (960 s). The random task phase contains four visual feedback tasks, and each task included one 60-s resting stage and three 60-s blocks (40 s for grasp and 20 s for rest over three blocks for a total of 1,200 s; Figure 3).

When the subjects performed different tasks, the corresponding cerebral cortex was activated. The increase in neuronal activity was accompanied by the increase in cerebral oxygen metabolism, and blood oxygen was consumed resulting in the changes in the concentrations of oxygenated hemoglobin (HbO_2_) and deoxygenated hemoglobin (HbR). The relative concentration change of the fNIRS data was calculated according to the modified Beer-Lambert law and then filtered by a Butterworth band-pass filter with a frequency band of 0.01-0.2 Hz. Previous studies have demonstrated that HbO_2_ was a reliable and sensitive indicator of movement-related changes in brain activation (Chen et al., 2020). Therefore, this study selected the concentration change of HbO_2_ (ΔHbO_2_) as the outcome measurement for analysis.

#### Regions of Interests

Regions of interest were set on bilateral primary sensorimotor cortex (SM1), pre-motor cortex (PMC), and dorsolateral prefrontal cortex (DLPFC) as shown in Figure 3. According to the results of MRIcro registration, the average value of all channels in the regions of interests greater than 50% was used as the activation value of the brain area (Ye et al., 2009; Wan et al., 2018). Performing the general linear model (GLM) and average beta values were obtained in the channel of the regions of interest (ROIs).

### Statistical Analysis

SPSS 25.0 software was used for statistical analysis. The measurement data conform to normal distribution and were expressed as the mean ± standard deviation; enumeration data were expressed as the rate or composition ratio. One-way analysis of variance was used to compare to measurement data between multiple groups in line with normal distribution and homogeneity of variance, and the Least Significant Difference method was used for pairwise comparisons between groups. The Kruskal–Wallis H method was used to compare the measurement data among multiple groups that did not meet the application conditions.

## Results

**FIGURE 4:**
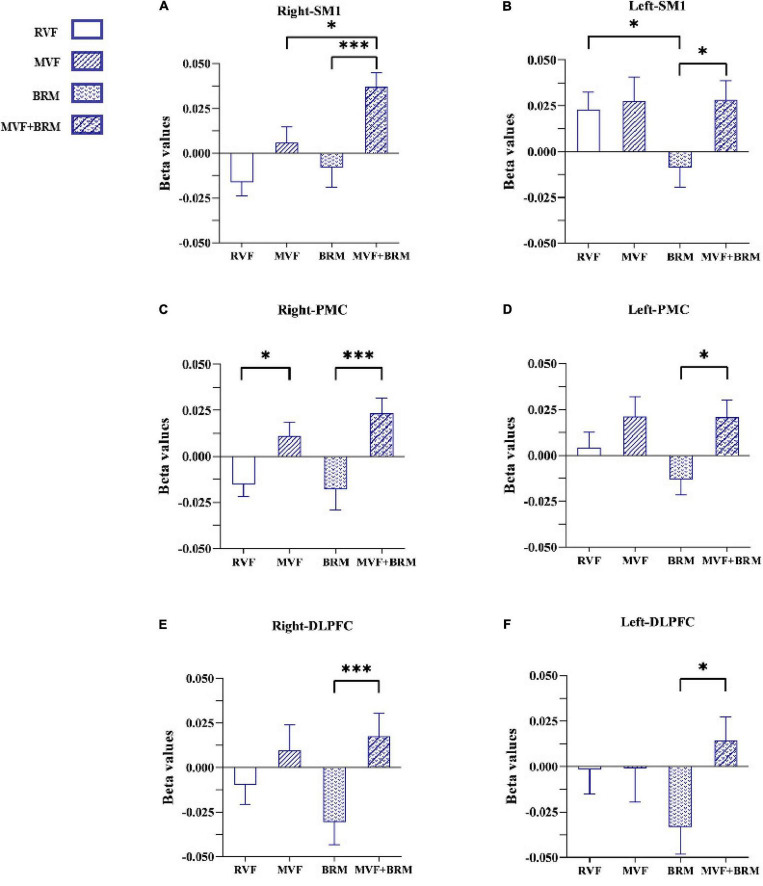
The comparisons of average beta values of ROIs in different four visual feedback tasks. **(A)** Right-SM1; **(B)** Left-SM1; **(C)** Right-PMC; **(D)** Left-PMC; **(E)** Right-DLPFC; **(F)** Left-DLPFC. Error bars represent standard error. *P < 0.05 and ^***^P < 0.001. RVF task, real visual feedback task; MVF task, mirror visual feedback task; BRM task, bilateral robotic movement task; MVF + BRM task, Mirror visual feedback combined with bilateral robotic movement task. SM1, primary sensorimotor cortex; PMC, pre-motor cortex; DLPFC, dorsolateral prefrontal cortex.

**FIGURE 5:**
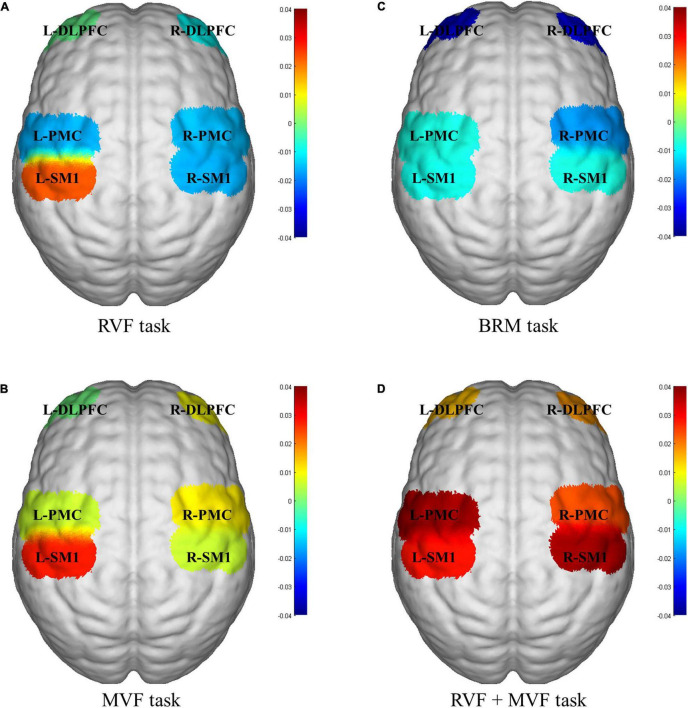
fNIRS activation maps for four visual feedback tasks. **(A)** RVF task; **(B)** MVF task; **(C)** BRM task; **(D)** RVF+MVF task. The beta values are indicated by color. RVF task, real visual feedback task; MVF task, mirror visual feedback task; BRM task, bilateral robotic movement task; MVF + BRM task, Mirror visual feedback combined with bilateral robotic movement task; SM1, primary sensorimotor cortex; PMC, pre-motor cortex; DLPFC, dorsolateral prefrontal cortex; L, left; R, right.

Twenty healthy subjects included in our study (nine males and eleven females; age: 22.150 ± 2.661 years). The statistical results showed that compared with RVF task, the beta value of the right PMC of MVF task (RVF task: -0.015 ± 0.029, MVF task: 0.011 ± 0.033, *P* = 0.033) was higher, and the difference was statistically significant. Compared with BRM task, the beta value of left SM1 in RVF task was higher (RVF task: 0.023 ± 0.042, BRM task: -0.001 ± 0.049, *P* = 0.047), and the difference was statistically significant. Compared with MVF task, the beta value right SM1 in MVF + BRM task was higher (MVF task: 0.006 ± 0.040, MVF + BRM task: 0.037 ± 0.036, *P* = 0.016), and the difference was statistically significant. Compared with BRM task, the beta values of right DLPFC (BRM task: −0.031 ± 0.057, MVF + BRM task: 0.018 ± 0.057, *P* = 0.009), right PMC (BRM task: −0.018 ± 0.050, MVF + BRM task: 0.024 ± 0.036, *P* = 0.001), right SM1 (BRM task: −0.008 ± 0.049, MVF + BRM task: 0.037 ± 0.036, *P* = 0.001), left DLPFC (BRM task: −0.033 ± 0.067, MVF + BRM task: 0.014 ± 0.058, *P* = 0.029), left PMC (BRM task: −0.013 ± 0.038, MVF + BRM task: 0.021 ± 0.042, *P* = 0.012), and left SM1 (BRM task: −0.009 ± 0.049, MVF + BRM task: 0.028 ± 0.047, *P* = 0.021) in MVF + BRM task were higher, and the difference was statistically significant (Figures 4, 5).

## Discussion

Our study used the synchronous fNIRS to compare the immediate hemodynamics cortical activation of four MVF tasks in healthy subjects. We explored the synergistic gain effect on cortical activation from MVF combined with a soft robotic bilateral hand rehabilitation system for the first time. Specifically, the combination of MVF and the robotic bilateral hand rehabilitation system was more conducive for cortical activation than either approach alone.

We first compared the RVF and MVF tasks. The results showed that MVF activated PMC on the mirror side. Its potential mechanism might be related to the key role of PMC in the mirror neuron network. MVF training is a rehabilitation method derived from the mirror neuron system (MNS) (Zhang et al., 2018). Mirror neurons both fire when an individual observes an action and when he/she performs a similar action (Ruggiero and Catmur, 2018).

Mirror visual feedback is a more vivid observation of movement mediated by mirrors. It promotes motor imagery and is further related to the rehabilitation of motor function (Ding et al., 2018). Meanwhile, the frontal-parietal MNS system is constructed by the parietal lobe, PMC, and the tail of the inferior frontal gyrus. It is an important neural network with mirror features (Rizzolatti and Craighero, 2004; Frenkel-Toledo et al., 2016). As one of the main components of the mirror neurons system, the activation of PMC could be seen both in action observation and action execution (Sun et al., 2018; Xu et al., 2019). Studies have shown that PMC is composed of interconnected regions in the primary motor cortex located in the frontal lobe of the brain (Fornia et al., 2020a). The upper motor neurons in the PMC can directly affect motor behavior via axons due to the extensive mutual connections with the primary motor cortex (Fornia et al., 2020b). Studies related to fMRI have also shown that motor imagery could enhance exercise preparation by increasing the activation of the premotor cortex (Bajaj et al., 2015a,b). Accordingly, MVF induced by a MVF task activated PMC through the components of motor imagery. These may be used as a key node in exercise preparation and can improve the neuroplasticity of the motor-related cortical areas on the mirror side.

The results also showed that the MVF + BRM task was more significantly activated in the SM1 region of mirror side than MVF task. The MVF + BRM task could significantly activate bilateral SM1, PMC, and DLPFC regions versus the BRM task. This suggests that MVF combined with BRM was more conducive to the activation of brain functional regions than MVF only or BRM only. The combination of these two strategies could lead to a synergistic gain effect. Previous studies showed that MVF induced the patient’s embodiment of the feedback limb images through mirror illusion (Ding et al., 2020). Combined visual and proprioception feedback could enhance the perception of embodiment (Wittkopf et al., 2017). MVF only emphasizes the embodied perception caused by visual feedback: The limited feedback on proprioception might limit the clinical utility of MVF (Feys et al., 2004). Thus, this study combined MVF and robotic bilateral hand movement to synchronize visual feedback and proprioceptive feedback, thus enhancing the overall embodied experience of the subjects. Moreover, about 40% of people with an acute right hemispheric and 20% of people with a left hemispheric stroke present a unilateral neglect (Ringman et al., 2004). In clinical applications, the increased attention of the affected limbs mediated by the illusion image from MVF may be beneficial to activate the movement network of the affected side.

Finally, we compared the RVF and BRM tasks. The results showed that robotic bilateral hand movement reduced activation of the left SM1. Clinical experiments also confirmed the effectiveness of bilateral training in improving upper limb motor function. Lee et al. (2017) included thirty stroke patients and found that the bilateral upper limb exercise training was more effective than conventional occupational therapy in improving upper limb function of stroke patients. Previous studies have also reported that MVF can be regarded as a special type of bilateral movement (Deconinck et al., 2015), however, compared with conventional bilateral exercise, MVF uses visual illusions to replace the actual movement of the affected side. The combination of MVF and robot linkage device just makes up for this shortcoming. The simultaneous movement of bilateral upper limbs generated by the robotic linkage device could reduce the interhemispheric inhibition, and the proprioceptive feedback of the affected side due to simultaneous movement of the bilateral upper limbs was also conducive to connections between motion control and primary motor cortex. This led to integration of interhemispheric sensory movements. This mechanism was critical for post-stroke patients especially for patients with moderate to severe upper limb motor dysfunction who might experience more brain activation inhibition from the healthy side.

Past meta-analysis of mirror therapy suggests that mirror therapy can be at least as an adjunct to conventional rehabilitation for post-stroke patients with upper limb motor dysfunction and activities of daily living (Thieme et al., 2018). However, there was lack of relevant mechanism research. In our study, the different tasks of the MVF combined robot glove showed a synergistic gain effect on the activation of the sensory motor zone. It might be a perfect combination with the bilateral movement caused by the linkage device of the robot glove and the MVF optical illusion to achieve the “central-peripheral-central” closed-loop central control is related. At the same time, the same direction bilateral upper limb movement under the linkage device can be used to improve the inhibition of the hemisphere after stroke, and the repetitive training of the contralateral side to drive the affected side is conducive to the remodeling of the cranial nerves. It is the “MVF combined with upper limb robot training” mediating the rehabilitation of upper limb motor function of patients after stroke and providing theoretical support, which can be further clinically studied in the future.

### Limitation

This was a pilot study of healthy young subjects. The heterogeneity between subjects was carefully controlled. The differences in the results might largely be due to the different settings used for the experiments. Future work will test this in diseased subjects. Neuroimaging is also needed to study activation of the whole brain of MVF combined with other rehabilitation strategies.

## Conclusion

Our study used the synchronous fNIRS to compare the immediate hemodynamics cortical activation of four visual feedback tasks in healthy subjects. The results showed the synergistic gain effect on cortical activation from MVF combined with a soft robotic bilateral hand rehabilitation system for the first time, which could be used to guide the clinical application and the future studies.

## Data Availability Statement

The raw data supporting the conclusions of this article will be made available by the authors, without undue reservation.

## Ethics Statement

The studies involving human participants were reviewed and approved by the Ethics Review Committee of The Fifth Affiliated Hospital of Guangzhou Medical University. The patients/participants provided their written informed consent to participate in this study.

## Author Contributions

QL, HO, and YQ designed the study. QL, YQ, and YXZ drafted the manuscript. YL, WL, and RD performed the data analysis. JL, AY, YY, and YJ collected the data. JZ, AC, YNZ, SH, and BW wrote sections of the manuscript. QL and HO approved the final version of the manuscript. All authors contributed to manuscript revision, read, and approved the submitted version.

## Conflict of Interest

The authors declare that the research was conducted in the absence of any commercial or financial relationships that could be construed as a potential conflict of interest.

## Publisher’s Note

All claims expressed in this article are solely those of the authors and do not necessarily represent those of their affiliated organizations, or those of the publisher, the editors and the reviewers. Any product that may be evaluated in this article, or claim that may be made by its manufacturer, is not guaranteed or endorsed by the publisher.

## Footnotes

1
https://www.chictr.org.cn
